# The clinical relevance of the Hippo pathway in pancreatic ductal adenocarcinoma

**DOI:** 10.1007/s00432-020-03427-z

**Published:** 2020-10-24

**Authors:** Richard Drexler, Mirco Küchler, Kim C. Wagner, Tim Reese, Bernd Feyerabend, Moritz Kleine, Karl J. Oldhafer

**Affiliations:** 1 Asklepios Campus Hamburg, Semmelweis University Budapest, Hamburg, Germany; 2 Division of HPB Surgery, Department of Surgery, Asklepios Hospital Barmbek, Hamburg, Germany; 3 MVZ Hanse Histologikum GmbH, Hamburg, Germany; 4 Department of General, Visceral and Transplant Surgery, Hannover Medical School, Hannover, Germany

**Keywords:** Hippo pathway, Pancreatic cancer, Metastasis, PDAC, Prognosis

## Abstract

**Purpose:**

The Hippo pathway has broadened in cancer research in the past decade and revealed itself to be an important driver for tumorigenesis and metastatic spread. In this study, we investigated the clinical relevance of the Hippo pathway with regard to metastatic invasion, patients’ outcome and histopathological features.

**Methods:**

Protein expression of components of the Hippo pathway were analyzed by immunohistochemistry (IHC) using paraffin-embedded tissue from 103 patients who had been diagnosed with pancreatic ductal adenocarcinoma and had undergone surgery. Results were correlated with clinicopathological data, disease-free and overall survival.

**Results:**

Immunohistochemistry studies in pancreatic tumour tissues revealed a significant upregulation of MST1, MST2, pLATS, pYAP and 14-3-3, representing the active Hippo pathway, in non-metastasized patients (*p* < 0.01). In turn, the pathway is more inactive in metastasized patients and relating liver metastases as LATS1, LATS2, YAP, transcriptional factors TEAD2 and TEAD3 were upregulated in these patients (*p* < 0.01). A higher pYAP expression was associated with a favorable OS and DFS.

**Conclusion:**

The Hippo pathway is inactive in metastasized patients releasing the pro-metastatic and proliferative potential of the pathway. Furthermore, our study underlines the prognostic relevance of the Hippo pathway as a shift in the balance towards the inactive pathway predicts an unfavorable OS and DFS.

## Introduction

Pancreatic ductal adenocarcinoma (PDAC) remains a challenging disease with a poor prognosis. The 5-year survival rate is between 4 and 8% with surgical resection remaining the only curative option (Ilic and Ilic 2016; Ferlay et al. 2013). At the time of diagnosis only 15–20% of patients are eligible for surgery and up to 50% of patients display hepatic metastasis (Siegel et al. 2018; Vincent et al. 2011). Ultimately 70% of the patients die from metastatic disease (Hogendorf et al. 2018; Ryan et al. 2014a, b).

The Hippo pathway consists of a large network of proteins, which control end organ size of different tissues, by regulating proliferation, cell growth, and apoptosis (Yu et al. 2015; Zhao et al. 2007; Saucedo and Edgar 2007). The pathway comprises of a core kinase cascade, starting with an activation of a pair of serine/threonine kinases mammalian STE20-like protein kinase (MST1/2), which activate another set of kinases, pair large tumour suppressor kinase (LATS). LATS1/2 phosphorylates the transcriptional activator Yes-associated protein (YAP), causing it to be transported from the nucleus to the cytoplasm. As a result, phosphorylated YAP (pYAP) accumulates with 14-3-3 protein, which causes cytoplasmatic sequestration (Boggiano et al. 2011; Poon et al. 2011; Glantschnig et al. 2002; Hergovich et al. 2006; Meng et al. 2015; Chan et al. 2005, p. 20). When the pathway is inactive, YAP can be found in the nucleus and interacts with transcription factors there, like TEAD 1–4 (Holden and Cunningham 2018; Lin et al. 2017a, b). The localization and phosphorylation of YAP are often used as a measure of Hippo pathway activity. Several studies prove that an overexpression of YAP is active in human cancer and successfully demonstrate that a higher expression or activity of YAP is linked with worse patient prognoses in various tumour entities (Wu et al. 2017; Poma et al. 2018; Yu et al. 2015; Harvey et al. 2013; Liu et al. 2018a, b; Zhang et al. 2015a, b; Zanconato et al. 2016). Furthermore, there is evidence that YAP is sufficient to drive cancer metastasis (Lamar et al. 2012; Nallet-Staub et al. 2014; Lau et al. 2014; Gu et al. 2016; Li et al. 2017, p. 1; Kim et al. 2017; Liu et al. 2016, p. 4, 2018a, b; Diepenbruck et al. 2014; Wang et al. 2018; Han et al. 2017, p. 16; Qiao et al. 2017; Zhou et al. 2016). In PDAC, the Hippo pathway has a pivotal role in disease progression, with formation of metastasis and YAP overexpression, which both correlate with an unfavorable OS (Xie et al. 2015; Salcedo Allende et al. 2017; Chen et al. 2015; Zhang et al. 2014). Here, we present the expression of all major proteins of the Hippo pathway in the largest trial population to date. The immunohistochemical results are linked with clinicopathological data including OS and DFS, which demonstrates the clinical impact of the pathway on patients with PDAC.

## Materials and methods

### Ethics approval

All patients’ data were fully anonymized, and the study was performed according to the standards set in the Declaration of Helsinki 1975. The tumour tissue used was remaining from material that initially had been collected for diagnostic purposes. All diagnostic procedures had already been fully completed when the samples were retrieved for the study. The study was approved by the Ethics Committee Hamburg, Germany (approval number PV5510).

### Patients’ characteristics

A total of 103 patients (female, *n* = 51; male, *n* = 52; median age, 67.8 years) diagnosed with PDAC, all of whom had undergone surgery between 2010 and 2018 at the Department of Surgery, Asklepios Hospital Barmbek-Hamburg (Germany) were included. The diagnosis was histologically confirmed and TNM classification was assessed according to the AJCC 7th edition. The R-status was obtained pathologically via the circumferential resection margin. All patients had a follow-up either up to their death (*n* = 76), or their most recent contact (*n* = 27) on June 30, 2020.

### Immunohistochemical analysis

Immunohistochemistry was employed to determine the intracellular localization and expression of all proteins. Immunohistochemical staining was performed using paraffin-embedded tissue. The tissue sections (4 µm) were deparaffinized in xylene and rehydrated in a descending alcohol set followed by heated antigen retrieval with 10 mM sodium citrate buffer (pH 6.0) or Tris–EDTA buffer (pH 8.0) for 5 or 30 min, respectively. Coverplates™ (ThermoFisher Scientific) were also used. Endogenous peroxidase activity was suppressed with Peroxide Block (Zytomed Systems). Primary monoclonal antibodies were diluted with Antibody Diluent (Zytomed Systems). Sections were covered with antibody and incubated at 4 °C for 24 h. Subsequently, ZytoChem Plus (HRP) Polymer Bulk Kit (Zytomed Systems) were used before staining with DAB (diaminobenzidin) Substrate Kit (Zytomed Systems). Gill’s hematoxylin III (Carl Roth) was used as a counterstaining agent, including a 10 s hydrochloric acid bath (5%) for differentiation. Sections were then dehydrated and mounted with EcoMount (Zytomed Systems).

Following primary antibodies were used: MST1 (1:150, Abcam (UK), ab51134), MST2 (1:100, Abcam (UK), ab52641), LATS1 (1:150, Abcam (UK), ab234820), LATS2 (1:50, Abcam (UK), ab135794), pLATS1 + 2 (1:50, Abcam (UK), ab111344), YAP (1:100, Abcam (UK), ab52771), pYAP-S127 (1:100, Abcam (UK), ab76252), 14-3-3-σ (1:50, Abcam (UK), ab14123), TEAD1 (1:500, Abcam (UK), ab133533), TEAD2 (1:75, Abcam (UK), ab196669), TEAD3 (1:500, Abcam (UK), ab237766) and TEAD4 (1:250, Abcam (UK), ab97460).

### Methods of evaluation

An immunoreactive score (IRS) was implemented for the evaluation of protein expression, which was based on the intensity and quantity of immune staining in the pancreatic cancer cells. The IRS score was applied as described by Kaemmerer and Remmele et al. (Kaemmerer et al. 2012; Remmele and Stegner 1987).

The intensity of staining was graded as negative (0), mild (1), moderate (2) and intense (3). The percentage of positive cells was evaluated as 0 (no positive cells), 1 (< 10% positive cells), 2 (10–50% positive cells), 3 (51–80% positive cells) and 4 (> 80% positive cells). The IRS score was obtained by multiplying these two individual scores. As a result, every tissue sample was classified into negative (IRS points 0–1), weak (2–3), mild (4–8) or strong (9–12). Two independent reviewers then evaluated the protein expression without prior knowledge of the patient characteristics.

### Statistical analysis

Differences in continuous variables were analyzed with the Mann–Whitney *U* test and differences in proportions were analyzed with the Chi-square test or Fisher exact test. Overall and disease-free survival was evaluated with the Kaplan–Meier method. Univariate and multivariate Cox proportional hazards models were used to assess the effects of variables on OS and DFS and to also compute mortality hazard ratios (HR). The Spearman rank order correlation was used for the pairwise correlation analyses of expression between proteins. A two-sided *p* value less than 0.05 was considered as statistically significant. All analyses were performed using SPSS Inc. (Chicago, IL, USA).

## Results

### Study population

A total of 103 patients who had been diagnosed with PDAC and undergone surgery between 2010 and 2018 were enrolled in this study. The patients had a median age of 67.8 years and 51 were female (49.5%). Features are listed in Table 1. Eight patients (7.8%) received neoadjuvant chemotherapy with FOLFIRINOX due to locally advanced PDAC. The majority of the tumours was located in the pancreas head (78.6%). Due to the most common tumour location, a pancreaticoduodenectomy (PDPP) was performed in the most cases (67.9%). Most patients suffered from a T3 stage (67.9%), nodal-positive (75.7%) and poorly differentiated (65.0%) tumour. The study population included patients in all possible variations of tumour stages and progression. Forty-two patients (40.8%) suffered from metastatic disease at the time of surgery. Of these metastasized patients, the majority presented with liver metastases (83.3%), while four patients had distant lymph node metastasis (9.5%) and three patients had peritoneal carcinomatosis (7.2%). Seven of these patients were evaluated as not resectable during surgery. After surgery, 88 patients (85.4%) were treated with adjuvant chemotherapy, mainly gemcitabine.

**Table 1:** A, B Correlation between tumour characteristics and IRS score of major components of the Hippo pathway

(A) Feature	*N*	MST1					MST2					LATS1					LATS2					pLATS				
	103	0	1	2	3	*p*	0	1	2	3	*p*	0	1	2	3	*p*	0	1	2	3	*p*	0	1	2	3	*p*
Gender																										
Female	51	13	14	17	7	0.68	8	8	26	9	0.82	4	18	20	9	0.27	17	13	19	2	0.22	15	12	18	6	0.61
Male	52	16	12	20	4		8	12	24	8		7	19	12	14		18	9	17	8		21	9	18	4	
Neoadjuvant chemotherapy																										
No	95	26	23	35	11	0.17	14	17	47	17	0.35	11	35	39	20	0.75	33	22	33	7	0.06	31	20	34	10	0.18
Yes	8	3	3	2	0		2	3	3	0		0	2	3	3		2	0	3	3		5	1	2	0	
Type of resection																										
PPDP	70	17	18	26	9	0.39	13	12	34	11	0.89	8	23	26	13	0.41	24	18	25	3	**< 0.01**	24	17	25	4	0.23
Left-sided resection	18	9	4	4	1		2	4	9	3		2	6	4	6		4	1	7	6		8	2	6	2	
Total pancreatectomy	15	3	4	7	1		1	4	7	3		1	8	2	4		7	3	4	1		4	2	5	4	
Tumour pathological stage																										
T1	8	4	0	4	0	0.55	0	3	2	3	0.35	0	3	4	1	0.47	4	0	3	1	0.33	2	0	4	2	0.11
T2	12	3	3	4	2		1	3	7	1		1	4	4	3		5	1	6	0		6	2	3	1	
T3	70	19	19	23	9		14	11	33	12		8	23	24	15		19	18	25	8		25	18	20	7	
T4	13	3	4	6	0		1	3	8	1		2	7	0	37		7	3	2	1		3	1	9	0	
Nodal status																										
N0	25	6	8	8	3	0.34	3	3	13	6	0.31	2	12	7	4	0.09	10	4	8	3	0.24	5	4	9	7	**< 0.01**
N1	70	18	16	28	8		12	13	35	10		8	25	23	14		23	18	22	7		24	17	26	3	
N2	8	5	2	1	0		1	4	2	1		1	0	2	5		2	0	6	0		7	0	1	0	
Metastatic status																										
M0	61	4	11	35	11	**< 0.01**	3	5	37	16	**< 0.01**	11	34	13	3	**< 0.01**	32	18	10	1	**< 0.01**	2	14	35	10	**< 0.01**
M1	42	25	15	2	0		13	15	13	1		0	3	19	20		3	4	26	9		34	7	1	0	
Tumour differentiation																										
Well-differentiated	7	2	0	3	2	0.13	0	1	5	1	0.89	1	4	2	0	0.21	5	1	1	0	0.42	1	3	2	1	**0.02**
Moderately differentiated	24	5	6	11	2		4	3	14	3		0	11	7	6		7	8	7	2		4	10	7	3	
Poorly differentiated	67	21	16	23	7		11	15	29	12		9	22	22	14		22	12	25	8		30	6	26	5	
Anaplastic	5	1	4	0	0		1	1	2	1		1	0	1	3		1	1	3	0		1	2	1	1	
Lymphatic invasion																										
L0	42	7	11	20	4	0.11	5	6	19	12	**0.04**	4	18	15	5	0.17	15	13	10	4	0.13	9	9	15	9	**< 0.01**
L1	61	22	15	17	7		11	14	31	5		7	19	17	18		20	9	26	6		27	12	21	1	
Perineural invasion																										
Pn0	10	3	1	5	1	0.65	1	1	6	2	0.78	1	5	2	2	0.78	5	3	1	1	0.36	2	3	3	2	0.48
Pn1	93	26	25	32	10		15	19	44	15		10	32	30	21		30	19	35	9		34	18	33	8	
Venous invasion																										
V0	50	14	12	19	5	0.97	7	8	27	8	0.72	5	19	17	9	0.74	18	13	12	7	0.09	14	11	22	3	0.16
V1	53	15	14	18	6		9	12	23	9		6	18	15	14		17	9	24	3		22	10	14	7	
Resection margin																										
R0	68	15	18	26	9	0.23	12	6	36	14	**< 0.01**	8	27	22	11	0.21	29	17	16	6	**< 0.01**	13	18	28	9	**< 0.01**
R1	35	14	8	11	2		4	14	14	3		3	10	10	12		6	5	20	4		23	3	8	1	
Tumour size [cm]^a^, median ± SMD: 3.5 ± 1.47																										
≤ 3.5	49	16	8	16	9	0.12	5	4	28	12	**< 0.01**	6	15	19	9	0.14	20	8	16	5	0.58	14	13	19	3	0.14
> 3.5	34	10	10	13	1		3	13	15	3		3	14	6	11		9	8	13	4		16	3	12	3	
Tumour localization																										
Head	81	18	20	32	11	0.21	13	16	37	15	0.81	10	29	27	15	0.45	30	20	28	3	**< 0.01**	26	19	29	7	0.36
Body	7	3	1	3	0		1	1	4	1		0	3	2	2		3	0	3	1		4	0	3	0	
Tail	11	6	3	2	0		2	1	7	1		1	4	3	3		2	1	5	3		3	2	4	2	
Body + tail	4	2	2	0	0		0	2	2	0		0	1	0	3		0	1	0	3		3	0	0	1	

(B) Feature	*N*	pYAP					YAP					14–3-3					TEAD1					TEAD2					TEAD3					TEAD4				
	103	0	1	2	3	*p*	0	1	2	3	*p*	0	1	2	3	*p*	0	1	2	3	*p*	0	1	2	3	*p*	0	1	2	3	*p*	0	1	2	3	*p*
Gender																																				
Female	51	15	8	15	13	0.61	16	13	17	5	0.49	11	7	28	5	0.31	16	21	13	0	0.58	15	15	13	8	0.29	15	11	24	1	0.26	17	20	13	1	0.47
Male	52	21	5	15	11		15	9	18	10		15	11	19	7		21	17	14	1		22	13	14	3		18	14	16	4		17	14	20	1	
Neoadjuvant chemotherapy																																				
No	95	30	12	30	23	0.07	29	21	34	11	**0.04**	21	17	46	11	0.11	32	37	25	1	0.46	37	25	24	9	0.18	31	23	37	4	0.76	33	31	30	1	0.15
Yes	8	6	1	0	1		2	1	1	4		5	1	1	1		5	1	2	0		0	3	3	2		2	2	3	1		1	3	3	1	
Type of resection																																				
PPDP	70	22	10	21	17	0.47	20	14	24	12	0.59	16	10	34	10	0.32	20	29	20	1	0.43	27	19	20	4	0.29	27	17	24	2	0.14	24	22	23	1	0.28
Left-sided resection	18	10	2	4	2		4	4	8	2		6	6	6	0		10	4	4	0		4	5	5	4		4	2	10	2		6	4	8	0	
Total pancreatectomy	15	4	1	5	5		7	4	3	1		4	2	7	2		7	6	3	0		6	4	2	3		2	6	6	1		4	8	2	1	
Tumour pathological stage																																				
T1	8	2	0	3	3	0.51	2	3	1	2	0.61	2	3	3	0	0.14	3	2	3	0	0.85	5	0	2	1	0.08	4	2	2	0	0.47	2	3	3	0	0.75
T2	12	5	2	2	3		3	3	3	3		4	0	8	0		3	7	2	0		5	5	2	0		3	3	6	0		2	4	6	0	
T3	70	27	9	18	16		21	12	28	9		18	14	30	8		26	23	20	1		19	20	23	8		20	15	30	5		25	23	21	1	
T4	13	2	2	7	2		5	4	3	1		2	1	6	4		5	6	2	0		8	3	0	2		6	5	2	0		5	4	3	1	
Nodal status																																				
N0	25	8	2	6	9	0.09	8	4	8	5	0.24	5	3	17	0	**0.04**	6	8	11	0	0.23	13	4	5	3	0.12	9	5	10	1	0.89	4	7	12	2	**0.03**
N1	70	24	8	24	14		22	15	26	7		17	15	28	10		29	25	15	1		23	22	17	8		21	19	27	3		26	26	18	0	
N2	8	4	3	0	1		1	3	1	3		4	0	2	2		2	5	1	0		1	2	5	0		3	1	3	1		4	1	3	0	
Metastatic status																																				
M0	61	8	3	27	23	**< 0.01**	29	15	13	4	**< 0.01**	3	9	37	12	**< 0.01**	26	23	12	0	0.12	31	17	11	2	**< 0.01**	26	19	16	0	**< 0.01**	23	23	14	1	0.12
M1	42	28	10	3	1		2	7	22	11		23	9	10	0		11	15	15	1		6	11	16	9		7	6	24	5		11	11	19	1	
Tumour differentiation																																				
Well-differentiated	7	1	0	3	3	0.53	2	1	2	2	0.84	1	2	4	0	0.64	3	3	1	0	0.61	5	1	1	0	**0.02**	5	1	1	0	0.34	2	1	3	1	0.24
Moderately differentiated	24	7	1	9	7		8	6	9	1		4	5	11	4		8	10	5	1		8	6	7	3		10	5	9	0		10	8	6	0	
Poorly differentiated	67	26	11	17	13		19	15	22	11		20	9	31	7		24	22	21	0		24	21	14	8		16	18	28	5		19	23	24	1	
Anaplastic	5	2	1	1	1		2	0	2	1		1	2	1	1		2	3	0	0		0	0	5	0		2	1	2	0		3	2	0	0	
Lymphatic invasion																																				
L0	42	8	4	15	15	**0.01**	11	12	10	9	0.09	7	7	24	4	0.22	13	17	12	0	0.67	17	12	8	5	0.58	15	10	16	1	0.75	10	15	15	2	0.16
L1	61	28	9	15	9		20	10	25	6		19	11	23	8		24	21	15	1		20	16	19	6		18	15	24	4		24	19	18	0	
Perineural invasion																																				
Pn0	10	2	2	5	1	0.29	4	1	1	4	0.06	2	1	6	1	0.81	3	4	3	0	0.96	6	2	2	0	0.34	5	3	2	0	0.42	4	3	3	0	0.94
Pn1	93	34	11	25	23		27	21	34	11		24	17	41	11		34	34	24	1		31	26	25	11		28	22	38	5		30	31	30	2	
Venous invasion																																				
V0	50	15	4	19	12	0.17	16	12	13	9	0.39	13	8	26	3	0.29	16	20	13	1	0.63	20	14	11	5	0.76	17	11	20	2	0.92	14	14	20	2	0.14
V1	53	21	9	11	12		15	10	22	6		13	10	21	9		21	18	14	0		17	14	16	6		16	14	20	3		20	20	13	0	
Resection margin																																				
R0	68	16	9	23	20	**< 0.01**	24	12	21	11	0.26	13	13	34	8	0.25	25	25	17	1	0.88	28	16	18	6	0.37	24	17	24	3	0.69	24	20	22	2	0.55
R1	35	20	4	7	4		7	10	14	4		13	5	13	4		12	13	10	0		9	12	9	5		9	8	16	2		10	14	11	0	
Tumour size [cm]^a^, median ± SMD: 3.5 ± 1.47																																				
≤ 3.5	49	13	8	14	14	0.44	15	10	16	8	0.86	11	8	26	4	0.11	18	14	17	0	0.17	15	16	9	9	0.11	13	16	19	1	0.25	16	11	20	2	0.18
> 3.5	34	14	3	10	7		9	9	12	4		9	7	10	8		13	15	6	0		11	8	13	2		12	6	13	3		11	14	9	0	
Tumour localization																																				
Head	81	24	12	25	20	0.23	26	18	25	12	0.24	18	12	39	12	0.45	23	34	23	1	0.26	31	23	21	6	0.16	28	23	27	3	0.12	27	27	26	1	0.09
Body	7	3	1	1	2		2	3	1	1		3	1	3	0		4	2	1	0		3	2	0	2		1	2	4	0		1	4	1	1	
Tail	11	7	0	4	0		3	1	5	2		3	4	4	0		7	1	3	0		2	1	5	3		4	0	6	1		6	2	3	0	
Body + tail	4	2	0	0	2		0	0	4	0		2	1	1	0		3	1	0	0		1	2	1	0		0	0	3	1		0	1	3	0	

IRS score: 0 negative, 1 weakly positive, 2 mildly positive, 3 strongly positive expression

^a^Data available for 83 patients (80.6%)

### Expression of kinases MST1/2, LATS1/2 and pLATS

The Hippo pathway comprises of a core kinase cascade, starting with the activation of a pair of MST1/2, which phosphorylate and activate another pair of kinases—LATS1/2. A positive expression of all kinases was found in the majority of cases: MST1 was found in 71.8%, MST2 in 84.5%, LATS1 in 89.3%, LATS2 in 66.0% and pLATS in 65.1%. MST1, MST2 and LATS1 expression was upregulated compared with the corresponding healthy pancreatic tissue (*p* < 0.01, Table 2). In contrast, pLATS was downregulated in cancer cells (*p* < 0.01, Table 2). The association between the expression of kinases and tumour characteristics revealed significant correlations in our study (Table 1). In metastasized patients we observed a significantly lower IRS score of MST1, MST2 and pLATS (*p* < 0.01), which represents the active conformation of the Hippo pathway. In contrast, LATS1 and LATS2 were upregulated in metastasized patients (*p* < 0.01).

**Table 2:** Overview of Hippo pathway proteins in PDAC, corresponding pancreatic tissue, liver metastasis and surrounding liver tissue of metastasized patients

Antibody	PDAC	Healthy pancreatic tissue	*p*	PDAC	Liver metastasis	*p*	Liver metastasis	Healthy liver tissue	*p*
MST1									
Negative	29	18	**< 0.01**	17	23	0.64	23	5	0.33
Weak	26	45		11	3		3	10	
Mild	37	35		1	2		2	8	
Strong	11	5		0	1		1	6	
MST2									
Negative	16	13	**< 0.01**	9	16	0.08	16	13	0.19
Weak	20	20		11	11		11	15	
Mild	50	64		9	1		1	0	
Strong	17	6		0	1		1	1	
LATS1									
Negative	11	1	**< 0.01**	2	1	0.89	1	1	0.83
Weak	37	34		6	7		7	8	
Mild	32	68		13	15		15	15	
Strong	23	0		8	6		6	5	
LATS2									
Negative	35	7	0.12	2	3	0.35	3	1	**< 0.01**
Weak	22	38		5	10		10	5	
Mild	36	54		18	15		15	22	
Strong	10	4		4	1		1	1	
pLATS									
Negative	30	6	**< 0.01**	23	23	0.58	23	10	0.08
Weak	12	25		6	3		3	7	
Mild	22	35		0	3		3	12	
Strong	10	8		0	0		0	0	
pYAP									
Negative	36	33	**< 0.01**	18	20	0.15	20	18	0.72
Weak	13	38		8	7		7	9	
Mild	28	26		2	2		2	2	
Strong	23	3		1	0		0	0	
YAP									
Negative	31	48	**< 0.01**	2	6	0.06	6	12	0.08
Weak	22	36		6	4		4	6	
Mild	35	19		15	13		13	11	
Strong	15	0		6	6		6	0	
14-3-3									
Negative	26	18	**< 0.01**	16	21	0.58	21	11	0.08
Weak	18	27		6	6		6	12	
Mild	47	49		7	2		2	6	
Strong	12	9		0	0		0	0	
TEAD1									
Negative	37	60	**< 0.01**	4	20	0.26	20	27	**< 0.01**
Weak	38	32		9	5		5	2	
Mild	27	11		15	3		3	0	
Strong	1	0		1	1		1	0	
TEAD2									
Negative	37	45	**< 0.01**	2	5	0.67	5	5	**< 0.01**
Weak	28	23		9	6		6	11	
Mild	27	30		11	15		15	12	
Strong	11	5		7	3		3	1	
TEAD3									
Negative	33	72	**< 0.01**	4	9	0.55	9	13	0.55
Weak	25	26		5	8		8	12	
Mild	40	5		16	11		11	4	
Strong	5	0		4	1		1	0	
TEAD4									
Negative	34	76	**0.02**	7	13	0.32	13	17	**0.03**
Weak	34	22		7	11		11	10	
Mild	33	5		15	5		5	2	
Strong	2	9		0	0		0	0	

Of the metastasized patients, 29 corresponding liver metastases were examined. We did not find any significant difference between the expression of all kinases in pancreatic cancer cells of the metastasized primary tumour and the cells in the corresponding metastasis (Table 2). Notably LATS2 was expressed more frequently in the healthy liver tissue compared to the pancreatic cancer cells within the liver parenchyma (< 0.01).

Alongside the metastatic status, MST2 IRS score was found to be significantly lower in tumours with lymphatic invasion (*p* = 0.04) and in tumours with a diameter larger than 3.5 cm (*p* < 0.01). Furthermore, a lower IRS score of pLATS was significantly correlated with lymphatic invasion, nodal-positive and more undifferentiated tumours (*p* < 0.01).

### Expression of YAP, pYAP and 14-3-3

**Fig. 1:**
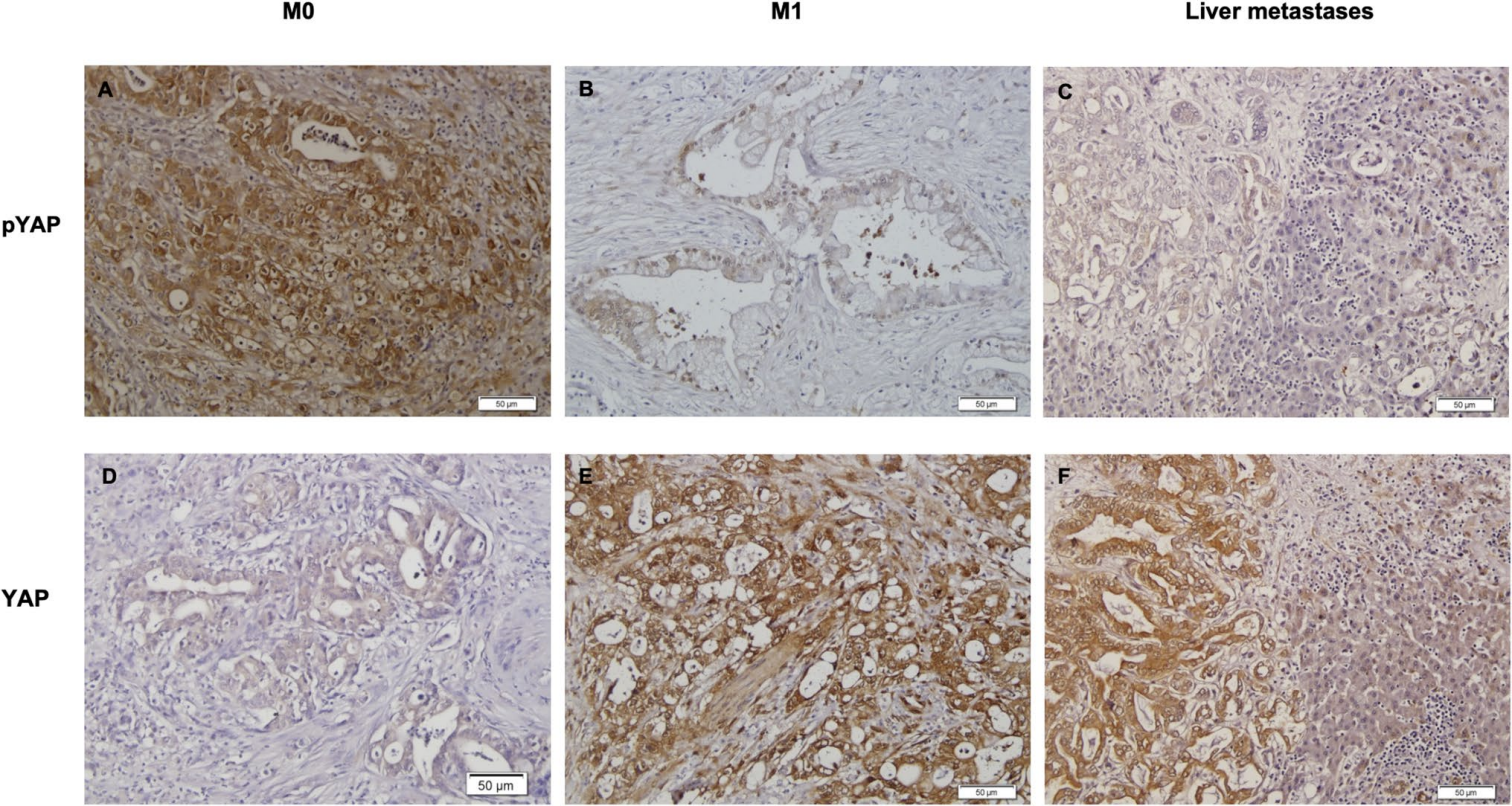
**a**–**f** Immunohistochemical staining of pYAP and YAP. Representative images of pYAP and YAP in non-metastasized (**a**, **d**) and metastasized patients (**b**, **e**) with their corresponding liver metastases (**c**, **f**). Scale bar, 50 μm

Expression of pYAP was detected in the cytoplasm, while YAP was mainly expressed in the nucleus of pancreatic cancer cells (Fig. 1). Both components, YAP and pYAP, were upregulated in PDAC compared to the corresponding healthy pancreatic tissue (*p* < 0.01). We observed a higher IRS score of YAP in patients receiving neoadjuvant chemotherapy (*p* < 0.04, Table 1B).

A lower expression of pYAP and the interacting 14-3-3 protein in pancreatic cancer cells was associated with the presence of metastases at time of surgery (*p* < 0.01, Table 1B). In contrast, YAP was more frequently expressed in these metastasized patients (*p* < 0.01, Table 1B). As already elucidated with the upstream kinases LATS1, LATS2 and pLATS, there was no significant difference regarding the expression of YAP, pYAP and 14-3-3 between the metastasized primary tumour and the relating liver metastases. Furthermore, the proteins had a similar expression in the surrounding liver parenchyma (Table 2).

### Expression of transcriptional factors TEAD1-4

In the inactive Hippo pathway, YAP is located in the nucleus and can interact with transcription factors TEAD1, TEAD2, TEAD3 and TEAD4 resulting in cell growth and proliferation. Therefore, we evaluated the nuclear expression of TEAD1, TEAD2, TEAD3 and TEAD4. All forms of TEAD were found to be upregulated in PDAC compared with corresponding healthy pancreatic tissue (Table 2).

A significantly higher TEAD2 and TEAD3 expression was observed in metastasized patients (*p* < 0.01, Table 1B). No significant associations were found for TEAD1, while TEAD4 was more frequently expressed in nodal-negative tumours (*p* = 0.03, Table 1B).

The liver metastases showed similar intensities of expression, as found in metastasized primary tumours. However, TEAD1, TEAD2 and TEAD4, but not TEAD3, were upregulated in the liver metastases in comparison with the respective surrounding liver parenchyma (Table 2).

### Activity of the Hippo pathway as an indicator for post-surgical prognosis

Data for overall survival was available for all 103 patients with 27 people (26.2%) living at the end of the study. Data regarding the time of recurrence was available to us for 84 of the patients and of these 84 patients, 56 patients (66.7%) suffered a recurrence. The majority (66.1%) had liver metastases. Seven patients suffered lung metastases (12.5%), five patients from peritoneal carcinomatosis (8.9%) and another seven patients from local recurrence (12.5%).

**Fig. 2:**
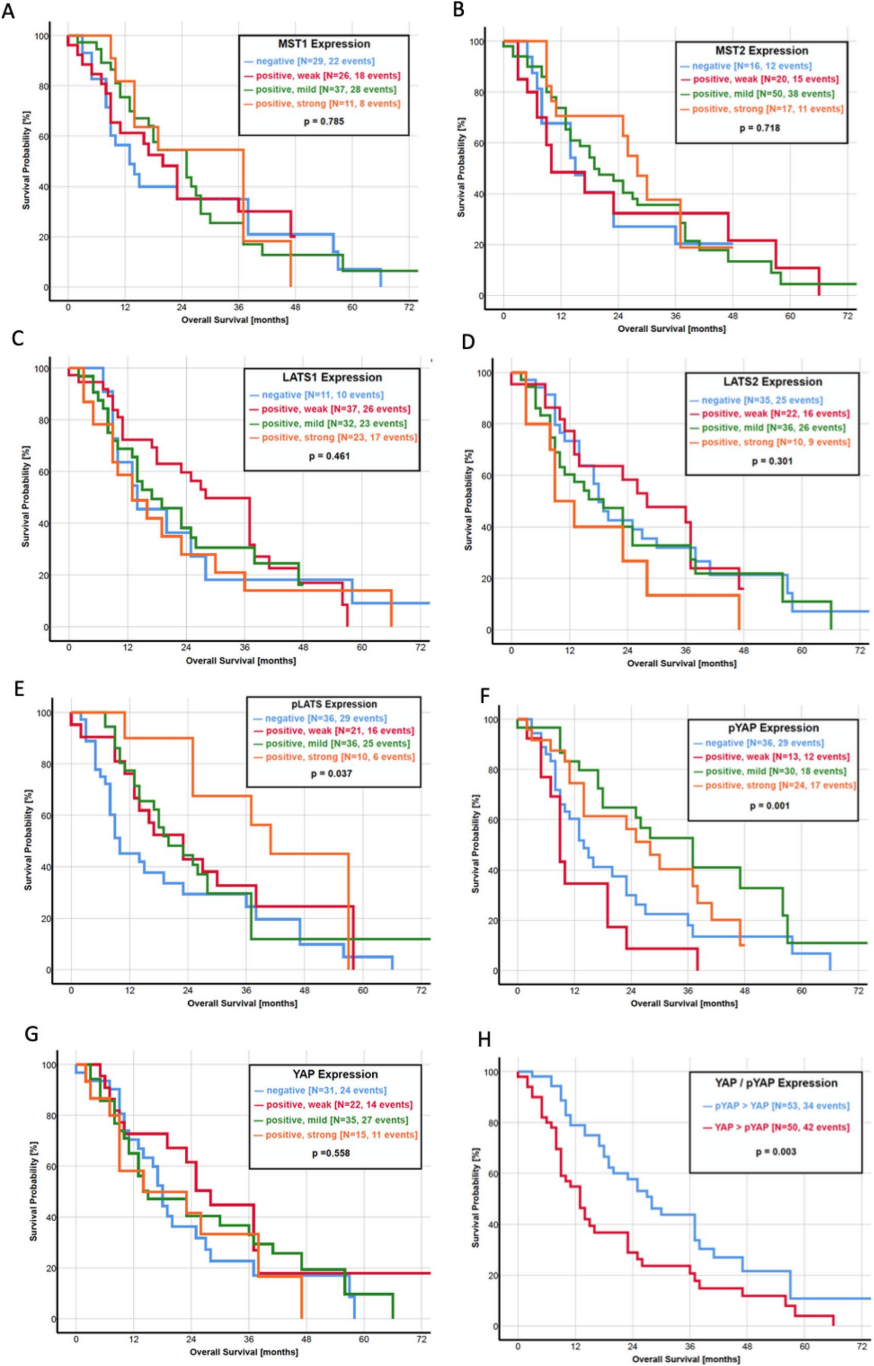

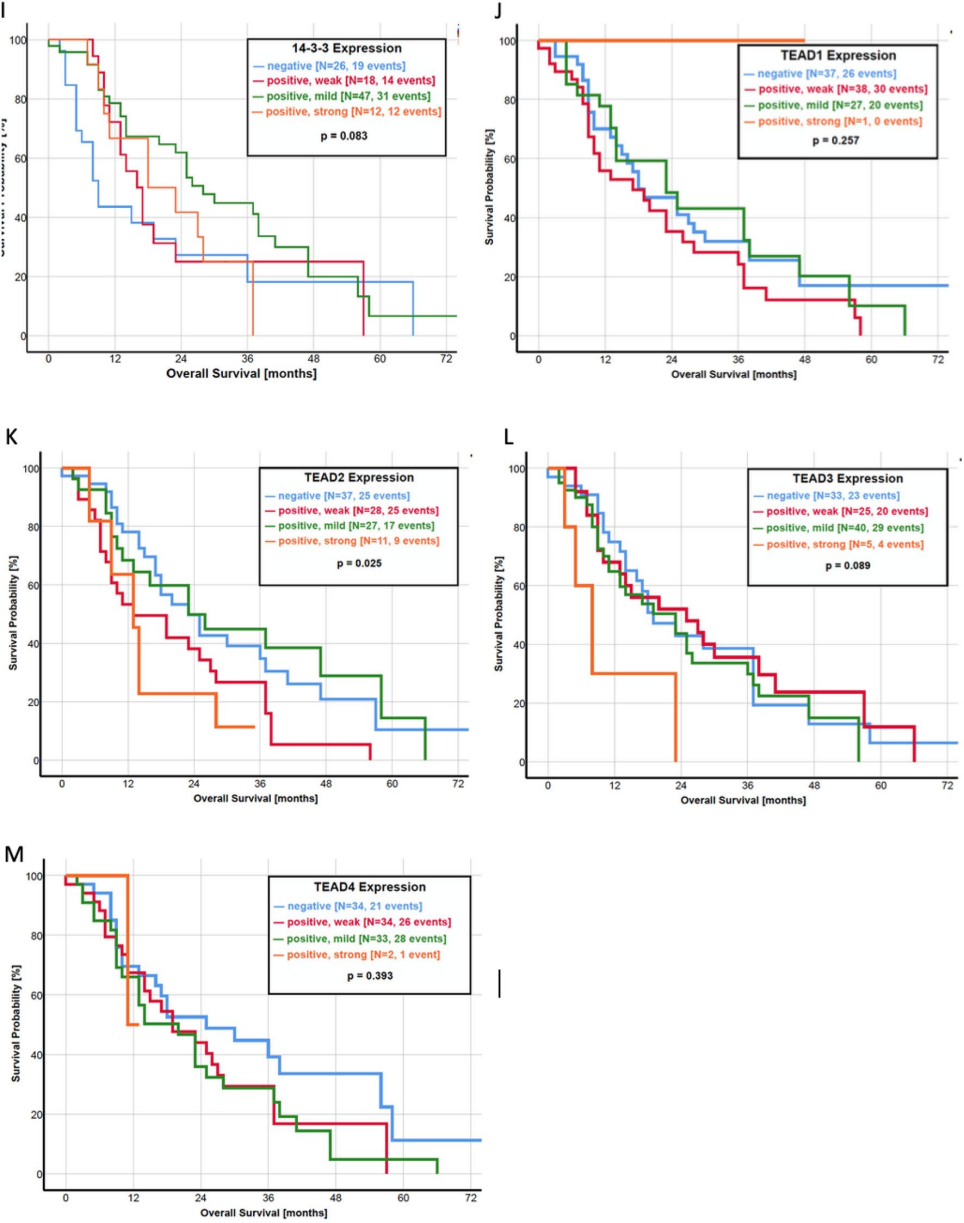
**a**–**m** Kaplan–Meier curves of overall survival for each protein of the Hippo pathway. **a** MST1, **b** MST2, **c** LATS1, **d** LATS2, **e** pLATS, **f** pYAP, **g** YAP, **h** ratio YAP/pYAP, **i** 14-3-3 protein, **j** TEAD1, **k** TEAD2, **l** TEAD3 and **m** TEAD4

Focusing on the association between the activity of the Hippo pathway and the survival of the patients, we created a ratio of YAP and pYAP, which compared the IRS score directly between both parameters and is also representative of pathway activity. A higher IRS score of YAP than pYAP (YAP > pYAP) resulted in a significantly shorter OS with a median survival of 13.0 months. In turn, a pYAP > YAP ratio was prognostically favorable with a median OS of 28.0 months (*p* = 0.003, Fig. 2h). Furthermore, patients with a mildly or strongly positive IRS score of pLATS (*p* = 0.037, Fig. 2e) and pYAP (*p* = 0.001, Fig. 2f) had a significant longer OS. In contrast, an upregulation of transcription factor TEAD2 corresponds to a worse prognosis regarding the OS (*p* = 0.025, Fig. 2k). However, the YAP expression itself was not significantly associated with the OS (*p* = 0.558, Fig. 2g). In a multivariate analysis, pYAP expression (HR: 0.51; 95% CI 0.22–2.01; *p* = 0.07) and metastatic status (HR: 0.47; 95% CI 0.18–1.22; *p* = 0.03) was associated with OS (Table 3).

**Table 3:** Cox proportional hazard for overall survival (*n* = 103)

Variable	No.	Median OS [months]	Univariate		Multivariate	
			Hazard ratio (95% CI)	*p* value	Hazard ratio (95% CI)	*p* value
Ratio pYAP/YAP expression						
pYAP > YAP	53	19.0	1.00		1.00	
YAP > pYAP	50	13.0	1.34 (0.86–2.11)	0.20	1.57 (0.53–4.71)	0.42
YAP expression						
Negative	31	17.0	1.00		1.00	
Positive, weak	22	24.0	0.66 (0.34–1.28)	0.22	0.34 (0.13–0.89)	0.78
Positive, mild	35	14.0	0.89 (0.51–1.56)	0.69	1.19 (0.16–1.54)	0.12
Positive, strong	15	13.0	1.01 (0.52–2.20)	0.84	1.03 (0.19–2.43)	0.49
pYAP expression						
Negative	36	13.0	1.00		1.00	
Positive, weak	13	9.0	1.79 (0.90–3.56)	0.09	1.27 (0.49–3.24)	0.62
Positive, mild	28	25.5	0.47 (0.26–0.84)	**0.01**	0.41 (0.17–0.99)	**0.04**
Positive, strong	23	21.0	0.69 (0.36–1.23)	0.19	1.42 (0.40–4.96)	0.59
Tumour pathological stage						
T1	8	19.0	1.00		1.00	
T2	12	10.0	1.66 (0.39–6.97)	0.49	0.94 (0.19–4.47)	0.94
T3	70	9.0	2.64 (0.83–8.46)	0.10	1.30 (0.37–4.62)	0.69
T4	13	13.0	2.74 (0.75–10.1)	0.13	2.14 (0.56–8.23)	0.27
Nodal status						
N0	25	23.0	1.00		1.00	
N1	70	16.0	1.74 (0.95–3.19)	0.08	1.90 (0.94–3.85)	0.13
N2	8	9.0	2.85 (0.91–8.98)	0.07	3.09 (0.84–11.4)	0.52
Metastasis status						
M0	61	20.0	1.00		1.00	
M1	42	11.5	1.66 (1.05–2.62)	**0.03**	2.19 (0.71–6.83)	0.17
Tumour differentiation						
Well-differentiated	7	33.0	1.00		1.00	
Moderately differentiated	24	20.0	2.19 (0.72–6.65)	0.17	2.06 (0.58–7.33)	0.26
Poorly differentiated	67	14.0	3.10 (1.10–8.65)	**0.03**	2.65 (0.84–8.39)	0.09
Anaplastic	5	16.0	1.96 (0.43–8.87)	0.38	1.51 (0.28–8.23	0.64
Resection margin						
R0	68	17.0	1.00		1.00	
R1	35	14.0	1.24 (0.77–2.01)	0.37	1.08 (0.61–1.92)	0.78

*CI* confidence interval

**Fig. 3:**
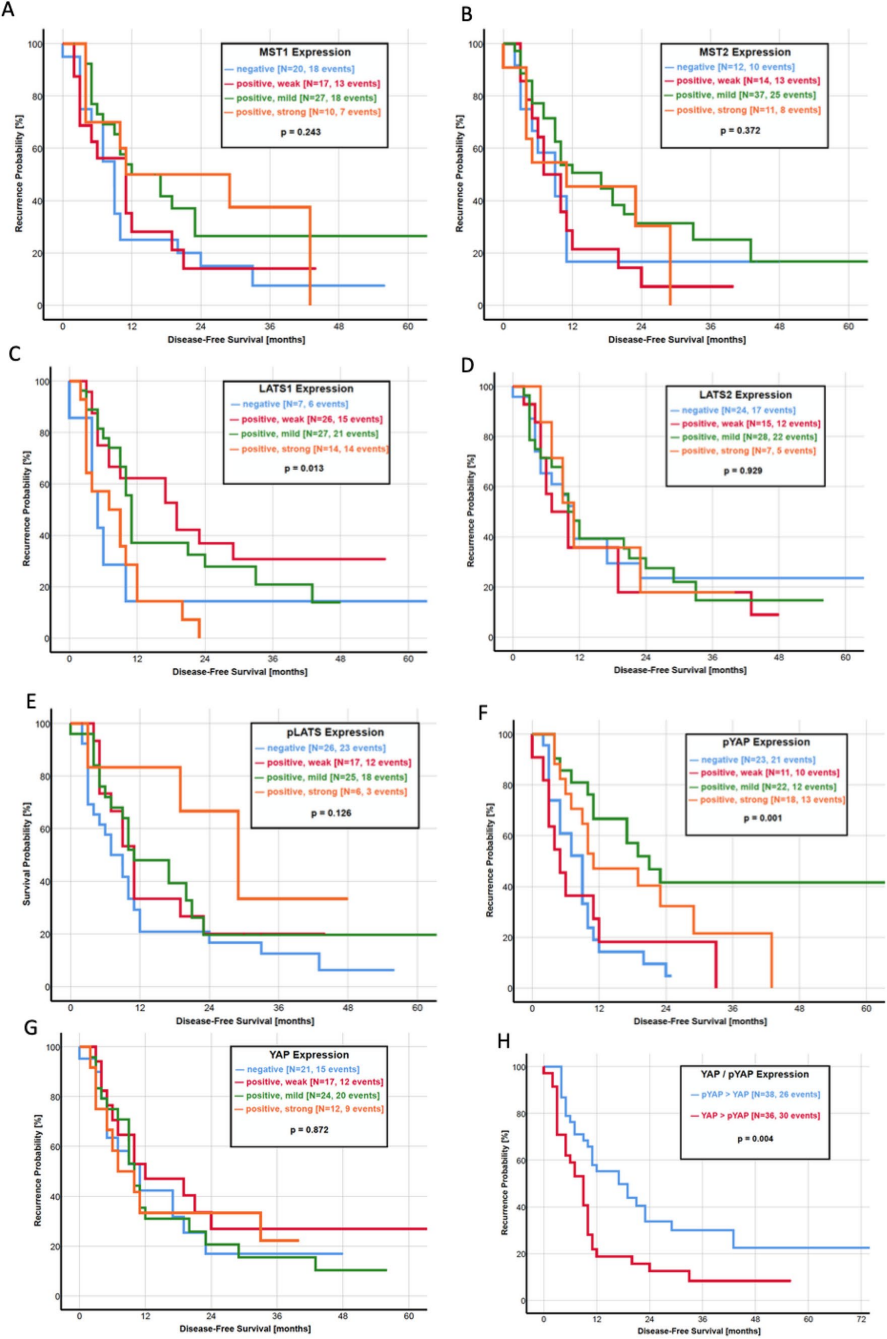

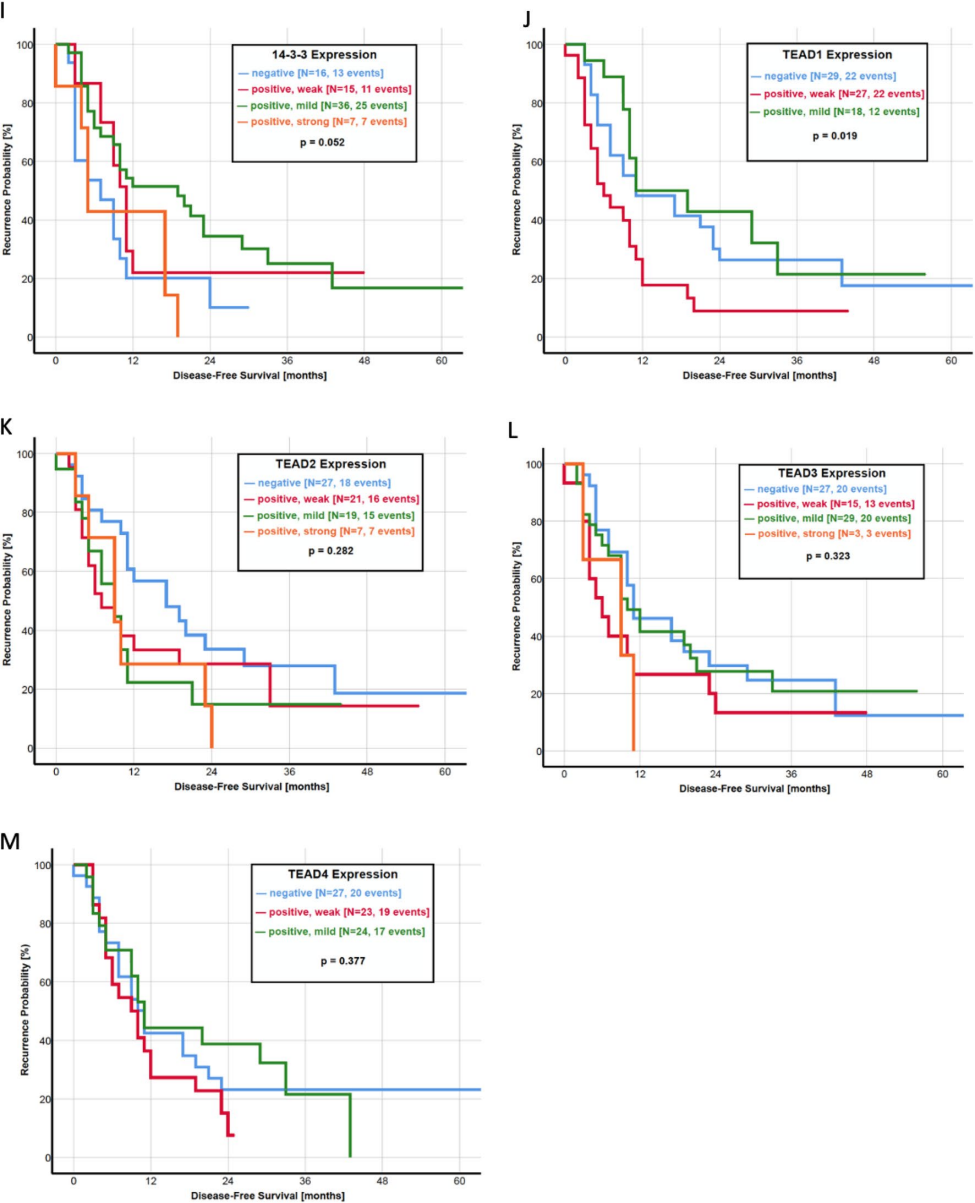
**a**–**n** Kaplan–Meier curves of disease-free survival for HSP27 and each protein of the Hippo pathway. **a** HSP27, **b** MST1, **c** MST2, **d** LATS1, **e** LATS2, **f** pLATS, **g** pYAP, **h** YAP, **i** ratio YAP/pYAP, **j** 14-3-3 protein, **k** TEAD1, **l** TEAD2, **m** TEAD3 and **n** TEAD4

The activity of the Hippo pathway seems to be an important factor in predicting the time until recurrence. As observed for the OS, a YAP > pYAP ratio was significantly associated with a shorter DFS than a pYAP > YAP ratio (*p* = 0.004, Fig. 3h). The median DFS in patients with a higher IRS score of YAP was 9.0 months, as compared with 17.0 months in patients with a pYAP > YAP ratio. In addition, a negative or weakly positive IRS score of YAP itself was correlated with a shorter DFS (*p* = 0.001, Fig. 3g). Of all other components of the Hippo pathway, only LATS1 (*p* = 0.013, Fig. 3d) and TEAD1 (*p* = 0.019, Fig. 3k) had a statistical significance in predicting time until recurrence.

Applying multivariate analysis, ratio YAP > pYAP (HR: 7.39; 95% CI 1.89–29.5; *p* = 0.005), YAP expression (HR: 0.2; 95% CI 0.04–0.96; *p* = 0.04), pYAP expression (HR: 5.55; 95% CI 1.09–28.4; *p* = 0.04), tumour pathological stage (HR: 3.23; 95% CI 1.57–6.63; *p* = 0.003) and nodal status (HR: 0.09; 95% CI 0.03–0.36; *p* = 0.001) was associated with DFS (Table 4).

**Table 4:** Cox proportional hazard model for disease-free survival (*n* = 75)

Variable	No.	Median DFS [months]	Univariate		Multivariate	
			Hazard ratio (95% CI)	*p* value	Hazard ratio (95% CI)	*p* value
Ratio pYAP/YAP expression						
pYAP > YAP	39	11.0	1.00		1.00	
YAP > pYAP	36	9.0	1.26 (0.75–2.14)	0.39	3.18 (0.62–16.3)	**0.02**
YAP expression						
Negative	21	9.0	1.00		1.00	
Positive, weak	17	12.0	0.77 (0.36–1.65)	0.49	0.57 (0.13–2.50)	0.45
Positive, mild	24	9.5	1.01 (0.52–1.98)	0.97	0.93 (0.17–5.24)	0.94
Positive, strong	13	10.0	1.71 (0.69–3.09)	0.12	1.92 (0.24–15.3)	**0.04**
pYAP expression						
Negative	24	8.5	1.00		1.00	
Positive, weak	11	5.0	1.01 (0.47–2.14)	0.99	0.33 (0.09–1.25)	0.10
Positive, mild	22	18.0	0.27 (0.13–0.57)	**< 0.01**	0.10 (0.03–0.35)	**< 0.01**
Positive, strong	18	10.5	0.48 (0.24–0.98)	**0.04**	0.27 (0.04–1.65)	0.16
Tumour pathological stage						
T1	7	19.0	1.00		1.00	
T2	11	10.0	2.69 (0.71–10.2)	0.14	1.83 (0.34–9.75)	0.48
T3	50	9.0	2.62 (0.81–8.50)	0.11	1.72 (0.44–6.70)	0.44
T4	7	13.0	3.83 (0.95–15.4)	0.06	6.08 (1.15–32.3)	**0.03**
Nodal status						
N0	18	18.0	1.00		1.00	
N1	49	10.0	1.63 (0.81–3.29)	0.17	2.03 (0.87–4.70)	0.10
N2	8	3.0	6.91 (2.60–18.3)	**< 0.01**	9.59 (2.52–36.5)	**< 0.01**
Metastasis status						
M0	44	11.0	1.00		1.00	
M1	31	9.0	1.62 (0.95–2.75)	0.07	1.17 (0.22–6.12)	0.85
Tumour differentiation						
Well-differentiated	4	32.5	1.00			
Moderately differentiated	18	11.5	6.31 (0.82–48.5)	0.07	6.92 (0.77–62.1)	0.08
Poorly differentiated	51	9.0	7.92 (1.08–58.2)	**0.04**	7.55 (0.89–64.4)	0.06
Anaplastic	2	22.0	3.42 (0.21–55.2)	0.39	9.04 (0.41–200.6)	0.16
Resection margin						
R0	50	10.0	1.00		1.00	
R1	25	10.0	1.09 (0.63–1.91)	0.74	1.77 (0.97–3.23)	0.08

*CI* confidence interval

## Discussion

PDAC is one of the most aggressive oncological diseases with limited therapeutic options for patients who often present with metastases at the time of diagnosis. This renders the prognosis by and large as unfavorable. Finding an efficient therapy to inhibit further metastases remains an almost insurmountable challenge but is necessary for improving patient survival. Therefore, understanding the molecular mechanisms that underlie metastatic processes is fundamental. In our study, we used immunohistochemical techniques to assess expression levels of the most important Hippo pathway components in 103 patients diagnosed with PDAC and treated with curative intention at our department. To the best of our knowledge, this is the largest cohort study investigating all major components of the Hippo pathway and correlating expression levels with clinicopathological results including OS and DFS.

The Hippo pathway is known for regulating cell proliferation, tissue homeostasis and organ size. Its role has broadened in cancer research in the past decade and it has been found to be a promoter of tumorigenesis and tumour migration (van Rensburg and Yang 2016; Han 2019; Moroishi et al. 2015; Lei et al. 2008). There is evidence that an inactivated pathway results in a higher nuclear YAP expression and in turn an unfavorable prognosis. All of this incurs a higher probability of spread of metastases in numerous malignancies (van Rensburg and Yang 2016; Zygulska et al. 2017; Yu et al. 2015; Harvey et al. 2013). Focusing on PDAC, Allende et al. associated a YAP overexpression with liver metastases and a poorer survival under 30 months in 64 curative treated patients (Salcedo Allende et al. 2017). Furthermore, different studies emphasize the importance of a YAP-driven cancer progression in PDAC in vitro and in vivo (Xie et al. 2015; Yang et al. 2015; Diep et al. 2012; Kapoor et al. 2014).

Returning to our study, we observed a significant upregulation of nearly all Hippo pathway components, except LATS2, in PDAC compared to healthy pancreatic tissue. Regarding the impact of the Hippo pathway upon tumour characteristics, we found a highly significant inactive shift in patients with metastases (Table 1). The proteins MST1, MST2, pLATS, pYAP and 14-3-3, representing the active pathway, were more frequently expressed in non-metastasized patients. In turn, we observed a significant upregulation of LATS1, LATS2 and YAP in patients suffering metastases. These results are comparable to previous findings by Allende et al. where YAP was found to be overexpressed in metastasized patients (Salcedo Allende et al. 2017). Our results confirm the involvement of the whole Hippo pathway in metastatic processes of PDAC and proves previous experimental results in a real-life cohort study (Xie et al. 2015; Yang et al. 2015; Wei et al. 2017; Yuan et al. 2016). In addition, we observed similar expression levels of all proteins in the relating liver metastases compared to their metastasized primary tumours. When comparing the expression of the metastasis with the surrounding liver tissue, we could not find a significant difference indicating a dysregulation of the Hippo pathway in the whole liver parenchyma. A supposed predisposition for more liver metastases growing but not detectable at time of surgery is hypothesized.

In the current literature, only three studies investigate the impact of Hippo pathway components on patient survival. Allende et al. found a significantly lower expression of YAP in patients with an OS under 30 months, but no differences in Kaplan–Meier analysis of OS and DFS (Salcedo Allende et al. 2017). A recently published study by Zhou et al. revealed YAP as an independent prognostic marker as a higher protein expression was associated with a shorter OS and DFS in 140 patients (Zhou et al. 2020). Another study led by Rozengurt et al. identified multiple YAP/TEAD-regulated genes as predictors with unfavorable survival by using the Human Protein Atlas (Rozengurt et al. 2018). As our study presents all of the important Hippo pathway components in the largest study population to this date, we correlated the expression of every single protein with the OS and DFS. Kaplan–Meier analysis revealed a favorable OS in patients with a higher expression of pLATS and pYAP (Fig. 2). In addition, patients with a higher expression of pYAP than YAP had a significantly longer OS. Almost similar results were observed regarding the DFS, where a higher pYAP expression and a pYAP > YAP ratio significantly correlated with a longer DFS (Fig. 3). These results underline the clinical importance of the Hippo pathway activity, measured by YAP and pYAP correlating to patient survival.

**Fig. 4:**
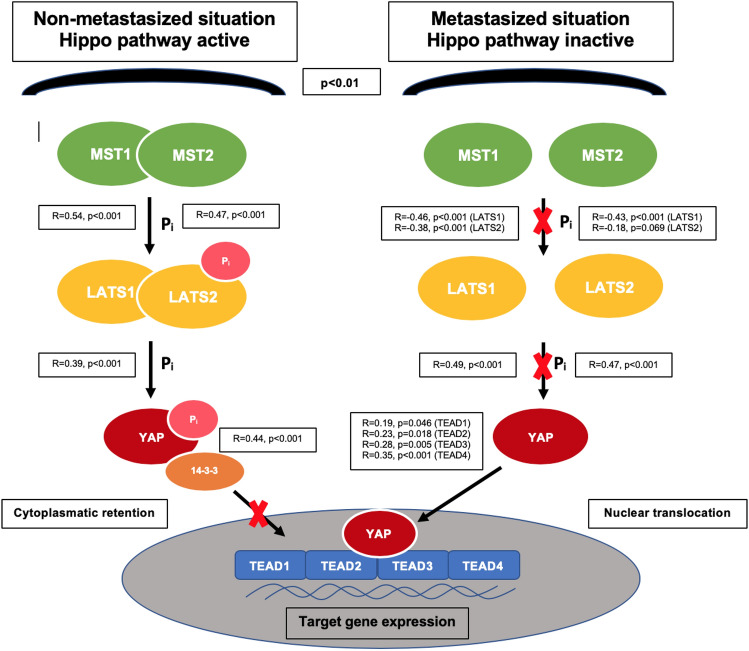
Illustration of the pathway in non-metastasized and metastasized patients according to our immunohistochemical results. *p* value refers to the difference of protein expression in regard to the metastatic status. Correlations are shown between expression of pathway components and their immediate downstream targets. Spearman rank order correlation was used for the pairwise correlation analyses

The effects of the Hippo pathway on proliferation, cell growth and homeostasis are mainly regulated by the nuclear transcription factors of the TEAD family including TEAD1, TEAD2, TEAD3 and TEAD4. It is widely accepted that TEAD takes plays a significant role in human cancer and the dissemination of cancer cells. However, TEAD activity and expression varies between different malignancies and has to be evaluated separately (Lamar et al. 2012; Holden and Cunningham 2018; Lin et al. 2017a, b; Huh et al. 2019). We therefore investigated the expression of each TEAD member to identify the major target of YAP after nuclear translocation in PDAC. Firstly, we found that all forms of TEAD were upregulated in PDAC compared with healthy pancreatic tissue. However, TEAD2 and TEAD3 were more frequently expressed in metastasized patients and their correlating liver metastasis, thus indicating that YAP promotes metastasis mainly through TEAD2 and TEAD3 (Fig. 4).

Focusing on prospective results, we envision huge potential in targeting the Hippo pathway to improve the prognosis of patients suffering from PDAC by preventing the metastatic spread or slowing down the rate of metastasis.

Numerous options of inhibiting the signaling pathway have been investigated as each component of the pathway could be potentially influenced. As there is evidence that crossover with other pathways exist, targeting YAP or the YAP-TEAD interaction as downstream effectors would be the preferable option (Holden and Cunningham 2018; Warren et al. 2018; Wu and Yang 2018). In addition, concentrating on YAP target genes is another promising approach as directly targeting YAP, e.g. with verteporfin. When further investigated, verteporfin was found to act as a competitor to TEAD binding site on YAP and is capable of disrupting the YAP-TEAD interaction (Liu-Chittenden et al. 2012). However, treatment with verteporfin is associated with substantial toxic side effects (Konstantinou et al. 2017; Zhang et al. 2015a, b). Nonetheless no active agents targeting YAP-driven genes promoting cancer growth, progression and metastasis have been approved for clinical use in PDAC, but it is a highly promising objective in the era of targeted therapy.

In conclusion, our study clearly shows that the Hippo pathway is inactive in metastasized patients resulting in nuclear translocation of YAP and an enhanced target gene expression via transcriptional factors TEAD2 and TEAD3 with pro-metastatic and proliferative effects. Furthermore, we revealed that the Hippo pathway has a huge impact on disease progression with metastatic spread and is clinically highly relevant as a shift in the balance towards the inactive pathway predicts an unfavorable OS and DFS. Therefore, we are confident that targeting the Hippo pathway could improve the outcome of patients suffering from PDAC and this role needs to be elucidated in prospective studies.

## Data Availability

All data generated that are relevant to the results presented in this article are included in this article. Other data that were not relevant for the results presented here are available from the corresponding author R. Drexler upon reasonable request.
